# Dynamics of Salivary Adenosine Deaminase, Haptoglobin, and Cortisol in Lipopolysaccharide-Challenged Growing Pigs

**DOI:** 10.3389/fvets.2021.698628

**Published:** 2021-10-14

**Authors:** Virpi Sali, Christina Veit, Anna Valros, Sami Junnikkala, Mari Heinonen, Janicke Nordgreen

**Affiliations:** ^1^Department of Production Animal Medicine, University of Helsinki, Mäntsälä, Finland; ^2^Department of Paraclinical Sciences, Norwegian University of Life Sciences, Oslo, Norway; ^3^Department of Production Animal Medicine, Research Centre for Animal Welfare, University of Helsinki, Mäntsälä, Finland; ^4^Department of Veterinary Biosciences, Faculty of Veterinary Medicine, University of Helsinki, Helsinki, Finland

**Keywords:** pig, LPS, ADA, haptoglobin, cortisol, saliva, experimental

## Abstract

Infectious and inflammatory conditions are common especially in growing pigs. Lipopolysaccharide (LPS) is an important antigenic structure of Gram-negative bacteria and can be used to induce inflammation experimentally. As pigs are usually group-housed in commercial conditions, it is difficult to detect sick individuals, particularly at an early stage of illness. Acute phase proteins such as haptoglobin (Hp) are known indicators of an activated innate immune system whereas adenosine deaminase (ADA) is a relatively novel inflammatory biomarker in pigs. Both parameters can be measured in saliva and could be used as indicators of inflammation. Compared with blood sampling, saliva sampling is a less stressful procedure that is rapid, non-invasive and easy to perform both at group and at individual level. In this blinded randomized clinical trial, 32 female pigs at their post-weaning phase were allocated to one of four treatments comprising two injections of the following substance combinations: saline-saline (SS), ketoprofen-saline (KS), saline-LPS (SL), and ketoprofen-LPS (KL). First, ketoprofen or saline was administered intramuscularly on average 1 h before either LPS or saline was given through an ear vein catheter. In all groups, saliva was collected prior to injections (baseline) and at 4, 24, 48, and 72 h post-injection for determination of ADA, Hp, and cortisol concentrations. A multivariate model was applied to describe the dynamics of each biomarker. Pairwise relationships between ADA, Hp, and cortisol responses from baseline to 4 h post-injection within the SL group were studied with Spearman correlations. A significant increase in the SL group was seen in all biomarkers 4 h post-injection compared to baseline and other time points (pairwise comparisons, *p* < 0.01 for all) and ketoprofen alleviated the LPS effect. We found a significant positive correlation between ADA and Hp within the SL group (*r* = 0.86, *p* < 0.05). The primary and novel findings of the present study are the response of ADA to LPS, its time course and alleviation by ketoprofen. Our results support the evidence that ADA and Hp can be used as inflammatory biomarkers in pigs. We suggest further studies to be conducted in commercial settings with larger sample sizes.

## Introduction

In commercial pig production, infectious and inflammatory conditions are common (1–3). Growing pigs are housed in groups of variable size, which potentially hinders the detection of sick individuals by herd employees. Moreover, sub-clinical illness poses a risk for disease transmission and can result in a reduced performance of pigs (4). In order to prevent disease outbreaks within herds and minimize production losses, it would be advantageous to detect problems as early as possible. Therefore, sampling methods that are easy to perform for a group of animals under practical farm conditions (4–6) would be of great value in pig herd health evaluation. Several biomarkers circulating in the bloodstream are detectable in saliva as well (6–9), and saliva sampling is also a less stressful alternative to blood sampling.

Lipopolysaccharide (LPS), also known as endotoxin, is an important antigenic structure of the cell wall in Gram-negative bacteria (10). It can be used experimentally to induce a systemic inflammation (11), which includes innate immune system activation (10) followed by an acute inflammatory response (10, 12) accompanied by sickness behavior (13). The key mediators during the inflammatory process are pro-inflammatory cytokines that trigger acute-phase protein (APP) production in the liver (12).

Haptoglobin (Hp) is an important APP in pigs (14, 15). It is primarily synthetized in the liver (12) yet some evidence about local Hp production in salivary gland exists (16). Serum Hp concentration is known to increase in pigs suffering from infectious diseases (5, 17, 18) or acute inflammatory processes (17, 18). Salivary Hp is elevated by systemic disease in pigs (19) and some evidence indicates that it is a more sensitive and specific biomarker for the detection of certain porcine diseases than serum Hp (9). It is also suitable for the detection of sub-clinical illness in pigs (5). Measurement of several APPs has been shown to improve diagnostic sensitivity (18), and increasing evidence supports determination of a panel of biomarkers with different triggers (7, 12, 17, 18) and dynamics (8, 20) instead of single ones.

Adenosine deaminase (ADA) is an enzyme involved in normal purine metabolism (21) and it is expressed in most tissues at some levels (22). Its expression, however, is highest in lymphoid organs indicating the role of ADA in immune activation (21) and ADA has been additionally proposed as a potential inflammatory biomarker in pigs (6, 19). Cortisol, which is usually perceived as a stress biomarker, is an indicator of activation of the hypothalamic-pituitary-adrenal (HPA) axis (8). Its release from the adrenal cortex happens within a few minutes under various stressful situations (8), including LPS injection (23), after which it is spread via the bloodstream.

Haptoglobin has been investigated previously in combination with ADA (6, 24) and cortisol (7, 20). To the authors' knowledge, neither the magnitude nor time course of the ADA response under a controlled immune challenge nor ADA's relation to Hp and cortisol in that setting have been described previously. Former reports have primarily been either cross-sectional (6, 19, 25) or longitudinal studies with sampling intervals of days or weeks (5, 7, 20, 24, 26) and conducted under farm conditions (5, 6, 24).

Ketoprofen is a commonly used non-steroidal anti-inflammatory drug (NSAID) in veterinary medicine and has been established as a potent anti-inflammatory drug in pigs (27, 28). NSAIDs target cyclo-oxygenase enzymes 1 and 2 (COX 1-2) and reduce pain, fever, and inflammation through inhibition of prostaglandin synthesis (29). Ketoprofen administration prior to LPS injection was recently shown to diminish the effect of LPS on cortisol and attenuated the behavioral signs of sickness in challenged pigs (30). An alleviating effect of an NSAID on an LPS-induced increase in one or more inflammatory biomarkers will strengthen the evidence for those biomarkers being sensitive indicators of pig health.

The aim of this experimental study was therefore to investigate the dynamics of salivary biomarkers of systemic inflammation in LPS – challenged growing pigs and to test whether an NSAID could alleviate the effect of LPS. We predicted that porcine salivary ADA, Hp, and cortisol would increase in response to LPS and that ketoprofen would alleviate the effect of LPS. In addition, we wanted to describe the correlations between the responses of salivary (1) ADA and Hp, (2) ADA and cortisol, and (3) Hp and cortisol in pigs injected with saline and LPS.

## Materials and Methods

### Ethical Statement

The Norwegian animal research authority approved the experiment (FOTS id 15232).

### Animals and Housing

The experiment took place in two blocks between April and May 2018 at the Livestock Production Research Center of the Norwegian University of Life Sciences (NMBU), campus Ås. Thirty-two female pigs (Norwegian Landrace), henceforth referred to as experimental pigs, were used in the study and comprised a subset of the pigs investigated by Veit et al. (30). The experimental pigs were 68–85 days (median 83 days) old at the beginning of the study. All pigs were kept in one room and group-housed in pens containing four experimental and two companion male pigs in order to increase the stocking density up to 1.3 m^2^ per pig. Pigs had visual and limited tactile contact with other pigs in the adjoining pen. One half of the pen (2.4 × 1.6 m) consisted of a solid lying area and the other half of slatted floor. Each pen had three nipple drinkers and pelleted feed (IDEAL S Die Ekstra, produced by Norgesfôr, Mysen, Norway) was provided for the pigs *ad libitum* at an animal-to-feeding place ratio of 3:1. The animal caretakers provided two handfuls of wood shavings and a handful of grass silage per pen on the lying area twice per day. Additionally, one handful of grass silage was placed in a rack. Each pen was equipped with a water sprinkler, which turned on every 10 min for 20 s. Lights were on between 6 am and 10 pm and the room was dimmed with night-lights during the night. Average ambient temperature in the unit was set to 20°C.

### Experimental Procedures

Within each pen, the four experimental pigs were randomly allocated to one of four treatments that were made up of four substance combinations: saline-saline (SS), ketoprofen-saline (KS), saline-LPS (SL), and ketoprofen-LPS (KL). The numbers of pigs per treatment were nine for SS and KL, and seven for KS and SL. The weight of the pigs was measured one the day before treatment and the pigs weighed between 16.3 and 50.7 kg (median 41 kg). The LPS dose used was determined according to previous research (23, 31) and for ketoprofen, the dosing was according to Fosse et al. (32). Ketoprofen (Romefen vet 100 mg/ml, Ceva Santé Animale, France) or saline were administered intramuscularly (i.m.) behind the ear. LPS (Serotype 0111: B4 of *Escherichia coli* dissolved in 0.9% sterile saline to a concentration of 100 μg/ml, produced by Sigma, Germany) or saline (sodium chloride 9 mg/ml) were administered intravenously (i.v.) through an ear vein catheter on average 61 ± 16 min after the first substance. The ear vein catheter was used only for injection, and removed immediately afterwards. The ketoprofen dose was 6 mg/kg, and the LPS dose 1.2 μg/kg. The pigs injected with LPS were observed closely in the hours after injection in order to detect individuals reacting stronger or for a longer time period than expected.

Repeated saliva samples were collected from individual pigs before any substance administration (baseline) and at 4, 24, 48, and 72 h after the intravenous injection. All baseline saliva samples were taken between 08:30 and 10:45 a.m. Each pig was allowed to chew a dental cotton pad suspended on a dental cord until it was moistened [modified from (33)]. Saliva was extracted by centrifuging the pad for 5 min at 1,000 × g. Saliva was pipetted to 2 ml Eppendorf tubes and stored on dry ice until it was moved to a −80°C freezer at the end of each sampling day.

### Salivary ADA, Hp, and Cortisol Measurements

Salivary ADA and Hp were measured in collaboration with a Spanish laboratory (Department of Animal Medicine and Surgery, Faculty of Veterinary Medicine, University of Murcia, Spain). A commercial automatized assay (Adenosine-Glutamate Dehydrogenase, BioSystems S.A., Barcelona, Spain) was used for ADA quantification according to the manufacturer's instructions. The method of the assay is based on the measurement of the decrease in absorbance (OD) per minute of a coupled reaction initially catalyzed by ADA (OD/min × 3,333 = U/L). The reaction is measured at 340 nm. Salivary Hp concentration was quantified by using an in-house time-resolved immunofluorometric assay, previously validated by Gutiérrez et al. (34). The assay is a non-competitive sandwich immunoassay based on the fluorescence of lanthanide chelate labels that provides a minimal background, lack of any sample interference, and an in-house highly specific monoclonal antibody against porcine Hp. Salivary cortisol concentration was measured using an enzyme immunoassay kit (DetectX®, Catalogue Number K0033-H5W, Arbor Assays, MI, USA) according to the manufacturer's protocol. Processing of saliva samples prior to cortisol analysis and the protocol itself are described in detail elsewhere (30).

### Statistical Analysis

SPSS (IBM SPSS Statistics 25) was used for statistical analysis of the data. Pig was used as experimental unit in all statistical analyses. In all statistical analyses, *p*-values below 0.05 were considered as significant and *p*-values of 0.05 ≤ 0.1 as tendency. Data normality was tested visually and with a Shapiro-Wilk test. Because none of the biomarkers studied met the normal distribution criteria, results are presented as median with range (see Table 1 in Results section).

**Table 1:** Median (min–max) values of adenosine deaminase (ADA), haptoglobin (Hp), and cortisol in saliva across 32 experimental pigs at different time points.

**Biomarker**	**Baseline** ***n* = 31**	**4 h p.i.** ***n* = 31**	**24 h p.i.** ***n* = 30**	**48 h p.i.** ***n* = 27**	**72 h p.i.** ***n* = 29**
ADA, U/L	118.0 (46.7–258.6)	115.3 (46.7–850.6)	137.0 (46.7–301.3)	112.7 (35.3–596.6)	108.7 (54.7–338.6)
Hp, μg/ml	0.33 (0.08–1.61)	0.31 (0.05–1.65)	0.45 (0.09–0.98)	0.37 (0.06–1.27)	0.23 (0.03–0.80)
Cortisol, ng/ml	0.32 (0.11–0.45)	0.33 (0.10–2.28)	0.30 (0.11–0.69)	0.22 (0.08–1.35)	0.20 (0.08–0.48)

*p.i., post-injection*.

To ensure normality of residuals and homogeneity of variance, all parameters were root-transformed prior to the statistical analysis. A multivariate approach was used to test the effect of LPS and ketoprofen on salivary ADA, Hp, and cortisol. Individual pigs were included as subjects and saliva sampling time point (0–72 h) as repeated measures. Saliva sampling time point and treatment and their interaction were added as fixed factors. Pre-planned pairwise comparisons were performed for all treatments at time point t4 and between different time points for the SL group using a Bonferroni correction. For the cortisol model, one pig belonging to the SS group was discarded from the analysis because it had exceptionally high salivary cortisol concentration at time point t4.

Non-parametric Spearman correlation was used to investigate whether ADA, Hp, and cortisol responses correlate between baseline (t0) and 4 h post-injection (t4). For this purpose, new outcome variables for each biomarker were generated for each individual in the SL group by calculating the difference in measured concentrations between time points t4 and t0.

## Results

### Dynamics of Salivary Biomarkers

**Figure 1:**
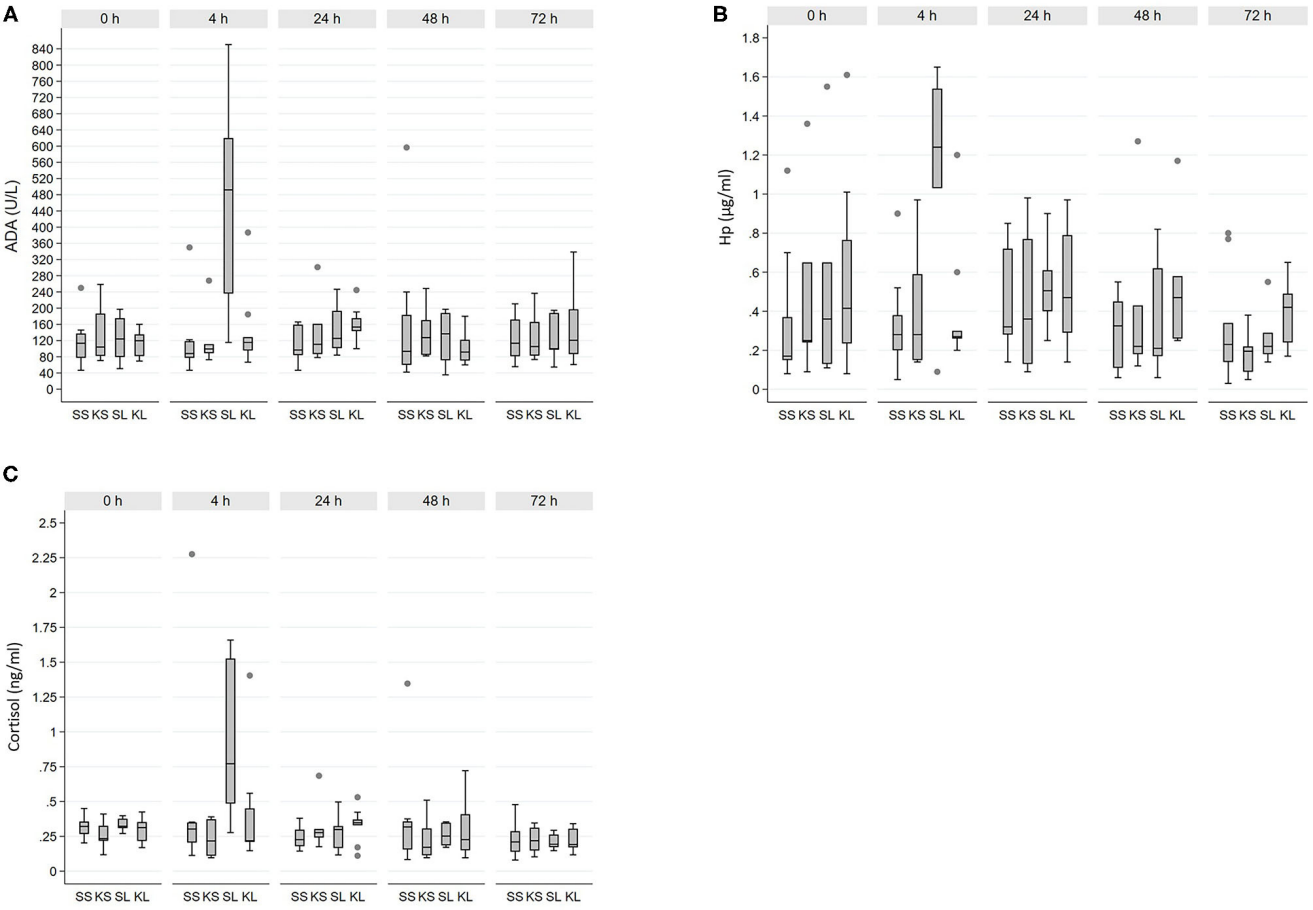
Raw values of salivary **(A)** adenosine deaminase (ADA), **(B)** haptoglobin (Hp), and **(C)** cortisol across 32 experimental pigs. SS, saline-saline; KS, ketoprofen-saline; SL, saline-LPS; KL, ketoprofen-LPS.

Descriptive results of salivary ADA, Hp, and cortisol measurements by sampling time point are shown in Table 1. Altogether seven ADA, seven Hp, and five cortisol samples were discarded from the analyses due to erroneous interpretation of the tube labeling in the laboratory. Raw values of each biomarker separated by time point are shown in Figure 1.

There was a significant interaction between time point and treatment for ADA (*F*_12, 58_ = 2.8, *p* = 0.01). ADA was clearly increased in SL 4 h post-injection compared with other treatment groups (pairwise comparisons, *p* < 0.01 for all). Moreover, ADA within the SL group at t4 was significantly increased relative to baseline and all other time points (pairwise comparisons, *p* < 0.001 for all). For Hp, the interaction between time point and treatment was not significant (*F*_12, 55.6_ = 1.7, *p* = 0.10). Overall, Hp concentration tended to be increased in the SL group compared with the SS group (pairwise comparisons, *p* = 0.06). However, 4 h post-injection Hp was significantly increased in the SL group compared with t48 and t72 (pairwise comparisons, *p* < 0.01 for both), but not with baseline or t24.

Cortisol response was similar to that of ADA and Hp, with a significant interaction between time point and treatment (*F*_12, 68.4_ = 1.9, *p* = 0.04). A significant increase in salivary cortisol concentration occurred at t4 in SL compared with SS and KS (pairwise comparison, *p* < 0.01 for both), and it tended to be higher than KL (pairwise comparison, *p* = 0.05). In the SL, salivary cortisol was significantly increased at t4 relative to baseline and all other time points (pairwise comparisons, *p* < 0.01 for all).

### Correlations Between Salivary Biomarkers

Across all experimental pigs, ADA, Hp, and cortisol did not correlate (Spearman correlation, *p* > 0.05 for all) at baseline. The response values calculated between baseline and t4 in the SL group showed a significant correlation for ADA and Hp (*r* = 0.86, *p* < 0.05). Although no significant correlations were found between ADA and cortisol or between Hp and cortisol, the correlation coefficients were moderate for both (*r* = 0.64 and *r* = 0.57, respectively).

## Discussion

As predicted, LPS injection resulted in an increase in salivary ADA and Hp as well as in salivary cortisol. A significant elevation in all studied biomarkers occurred at 4 h post-injection in LPS-challenged pigs. In other treatment groups, including the KL group, their concentrations remained relatively stable during the study period. Based on this, pre-treatment with ketoprofen was able to alleviate the LPS effect. The response of ADA and Hp showed positive significant correlations in the SL group indicating their parallel dynamics under the influence of bacterial LPS.

Increased salivary ADA concentrations have been reported in pigs suffering from clinically evident infectious and inflammatory conditions (6) and in stressed sheep (35). Several pig studies have investigated concentrations of Hp induced by viral (5, 9, 17, 18, 36) or bacterial (17, 18, 37) pathogens. Stressful occasions can increase serum Hp concentration (38, 39) as well. In this experiment, basal Hp concentration in saliva varied considerably between individual pigs with a range of: 0.08–1.61 μg/ml. For serum, high inter-individual variation has been reported previously (40–42). As a strong positive correlation between serum and salivary Hp concentrations exists (5), serum and salivary Hp dynamics are comparable to each other. Moreover, we observed high Hp concentration in one pig per treatment group at baseline. All of these pigs were among the pigs of lowest weight within the respective groups. Gutierrez et al. (6) did not find an increase in salivary Hp, ADA in growth retarded pigs. Even though we did not examine the study pigs clinically, all experimental pigs appeared healthy both prior to and during the experiment. There might be, however, differences in stress responsivity or subclinical conditions that could explain our findings.

Administration of *E. coli* LPS mimics an endotoxemic state that is known to induce a systemic inflammatory response (43). The pigs in this study were a subset of those in Veit et al. (30), where the behavioral signs of illness were reported. While the clinical onset of acute inflammatory response was not confirmed [for details, see (30)], an earlier report with the same *E. coli* strain and LPS dose indicates a strong activation of the innate immune system already 1 h after LPS injection (23). The rapid increase in the concentrations of all biomarkers in this paper is in line with this. The concentrations of all biomarkers returned to baseline levels by 24 h post-injection. To the best knowledge of the authors, previous pig studies have not investigated either short-term dynamics of salivary ADA, or ADA concentrations of pigs under a controlled immune challenge.

Previous research has shown that when triggered by an infectious agent serum Hp remains high for several days in pigs (18, 20, 44, 45). Heegaard et al. (18) reported differing dynamics of serum Hp depending on the disease causative agent, including bacterial, viral, and parasitic ones, and compared with aseptic inflammation. The rapid decline in salivary Hp in the present study might have been caused by the use of a single low-dose of synthetically purified LPS, which was likely to be eliminated from the body faster than LPS during natural infection. Escribano et al. (20) reported a three-fold increase in salivary Hp after LPS treatment, which remained high throughout the 7-day study period in growing pigs. In contrast to our study, they used a different E. coli strain (O55:B5), about 30 times higher LPS dose and repeated LPS injections (20). Moreover, the LPS dose was raised between the consecutive injections (20). As predicted, salivary cortisol of LPS-injected pigs not pre-treated with ketoprofen peaked at 4 h after injection, confirming the findings of others measuring cortisol from saliva and serum (23, 30, 45, 46). These results are in line with those of Escribano et al. (20) and Nordgreen et al. (23), who reported that salivary and plasma cortisol was elevated for only a short period of time following LPS challenge.

Our results indicated that intramuscularly administered ketoprofen was able to inhibit the effect of LPS, when given 1 h prior to LPS injection. Others have shown a similar effect of orally administered ketoprofen pigs (27). Moreover, the bioavailability of oral and intramuscularly administered ketoprofen has been reported to be similar (28). The appropriate dose of oral ketoprofen was set at 2 mg/kg (27), which is a third of the dose administered to KS and KL pigs in the present study. Mustonen et al. (46) reported that the effect of oral ketoprofen was seen immediately after its administration and that the effect lasted for ~7 h.

Because the concentration of all biomarkers peaked at the same time point in the SL group, we wanted to test whether the increases from baseline to 4 h post injection were correlated. A significant correlation was found only between ADA and Hp. Gutiérrez et al. (25) reported a significant positive correlation between salivary ADA and Hp in healthy finishing pigs. In addition, their study population contained both female and male pigs (25) and therefore the comparison between the results of these two studies is not straightforward. Neither ADA nor Hp correlated with cortisol in the SL group. Contreras-Aguilar et al. (35) reported a significant correlation between salivary ADA and cortisol concentrations in sheep caused by either shearing stress or being frightened by a dog. Although we found no significant correlations between these, the correlation coefficients were at least moderate compared with Contreras-Aguilar et al. (35), who reported low correlation coefficients (0.34 and 0.19, respectively).

The present study was conducted in experimental conditions, with a possibility to optimize the management and housing conditions of the experimental pigs. The sampling occurred in pre-defined times during each day in order to avoid potential bias caused by a circadian rhythm as reported for cortisol (47) and Hp (48). To the best of our knowledge, no circadian pattern has been reported for ADA. The experimental pigs were of same age, breed and sex thus the potential bias caused by those factors (24) was supposed to be negligible. However, further studies including both genders as well as pigs at different stages of production should be conducted to investigate the dynamics of salivary biomarkers more thoroughly and to extrapolate the results to be applied in commercial settings.

## Conclusions

The salivary concentration of ADA, Hp and cortisol increased rapidly after LPS challenge and they followed a similar pattern, and ketoprofen was able to alleviate the LPS effect. The results indicate that the selected salivary parameters, are indicative of systemic inflammatory response in pigs at an early stage. Primary and novel findings of the study are the response of ADA to LPS, its time course, and alleviation by ketoprofen. The usefulness of these biomarkers should be validated in a larger sample and in practical farm conditions.

## Data Availability Statement

The raw data supporting the conclusions of this article will be made available by the authors, without undue reservation.

## Ethics Statement

The animal study was reviewed and approved by the Norwegian Animal Research Authority (FOTS id 15232).

## Author Contributions

VS drafted the manuscript, prepared the raw data, performed statistical analyses, and assisted in sample collection. CV processed the manuscript together with VS and participated in planning the experiment and sample collection. JN planned the experiment and performed sample collection. AV participated in experiment planning and statistical analyses. JN, MH, and SJ were actively involved in the manuscript writing process together with VS, CV, and AV. All authors contributed to the article and approved the submitted version.

## Funding

VS received funding for the ADA and Hp analyses from the Veterinary Research Foundation (ETTS). The research visit by VS to NMBU was funded by a Short-term Scientific Mission awarded by the COST ACTION Grouphousenet (CA15134). COST was supported by the EU Framework programme Horizon 2020. The experiment was funded by the Norwegian University of Life Sciences.

## Conflict of Interest

The authors declare that the research was conducted in the absence of any commercial or financial relationships that could be construed as a potential conflict of interest.

## Publisher's Note

All claims expressed in this article are solely those of the authors and do not necessarily represent those of their affiliated organizations, or those of the publisher, the editors and the reviewers. Any product that may be evaluated in this article, or claim that may be made by its manufacturer, is not guaranteed or endorsed by the publisher.
